# Comprehensive Assessment of Ultrasound’s Role in Carpal Tunnel Syndrome in Reference to Electromyography

**DOI:** 10.7759/cureus.20798

**Published:** 2021-12-29

**Authors:** Yaman M. Alahmad, Fatima Al-Khafaji, Mohamad Alhuda Mohamad Alahmad, Alaa Al-Taie

**Affiliations:** 1 Radiology, Hamad General Hospital, Doha, QAT; 2 Internal Medicine, Kansas City University, Kansas, USA

**Keywords:** diagnosis, sensitivity, ultrasound, cross-sectional area, carpal tunnel syndrome

## Abstract

Carpal tunnel syndrome (CTS) is the most common peripheral mononeuropathy related to entrapment syndrome. An increase in the median nerve cross-sectional area (CSA) is visualized using ultrasound (US) imaging for CTS patients. This study aims to correlate, for the first time in the state of Qatar, the findings of US imaging and electromyography (EMG) in patients with CTS for diagnostic and classification purposes. First, US CSA was numerically obtained from patients' records. Second, EMG findings were gathered as normal, mild, moderate, and severe CTS. After that, we performed a different statistical approach than those used in the literature, using one-way analysis of variance (ANOVA) and post hoc tests for the final analysis. In summary, we found that the US seems unable to differentiate some normal from mild CTS cases; however, it appears to be excellent at differentiating moderate from severe CTS cases.

## Introduction

Ultrasonography (US) is a continuously advancing radiological modality that uses sound waves and acoustic phenomena of body tissues to display an image of internal structures. Its ionizing radiation-free mechanism of action, non-invasiveness, low cost, and time-effectiveness have made it one of the most desired diagnostic tools for clinicians in daily practice. This has led to exponential growth in exploring its diagnostic profile in various medical conditions, including carpal tunnel syndrome (CTS).

CTS, the most common mononeuropathy, refers to the clinical features related to the compression of the median nerve at either wrist within the carpal tunnels. Even though the etiology behind CTS is largely unknown, several risk factors have been associated with it, such as female gender, obesity, diabetes, and hypothyroidism [1]. The hallmark feature of CTS is numbness or pain sensations distributed along with the median nerve territory over the wrist and hand. Although CTS is a clinical diagnosis, a confirmatory test is usually needed for uncertain cases. Electrodiagnostic studies, nerve conduction studies (NCS), and electromyography (EMG) are currently the gold standard for CTS evaluation. These studies carry a critical role in decisions regarding surgical intervention based on the severity of median nerve compression. US is another tool that can be used in CTS evaluation. An increase in the median nerve cross-sectional area (CSA) is seen using US imaging for CTS patients. Several studies suggest that the optimal cutoffs for diagnosing CTS are 8.5 mm2 to 10.0 mm2 [2,3]. However, there is a scarcity of data on the cutoffs for the CTS classification of severity indices (mild, moderate, and severe) using the US [4]. Identifying the severity of entrapment based on the US cross-sectional area of the median nerve may help radiologists classify cases during reporting; thus, it could influence the management and follow-up plan. Further, establishing relevant outcomes may contribute to the generalizability and external validity of previously published studies.

In Qatar, Hamad Medical Corporation (HMC), the primary governmental health care provider, has a significant number of patients who have been noted to have wrist US and EMG for evaluation of CTS. For the first time in Qatar, we aimed to study the correlation between the findings of US imaging and EMG in patients with CTS for diagnostic and classification purposes.

## Materials and methods

Study design and data collection

We conducted a retrospective cross-sectional study for the period between 2014 and 2020 to identify patients who are older than 18 years of age and had both US and EMG studies while attending/following up under HMC care in Doha, Qatar.

In HMC, US studies are performed by musculoskeletal radiologists using high-resolution linear array transducers in the evaluation of CTS. Patients are requested to sit comfortably while extending their arms and wrists over the examination table. The forearms are positioned supine and the fingers are semi-extended. The CSA of the median nerve was measured at the level of the proximal inlet of the carpal tunnel, corresponding to the anatomical landmark during US, which is the volar lunate convex surface. All US images are stored in the Radiology Information System/Picture Archiving and Communication System (RIS/PACS).

A randomized sample of 231 subjects was obtained from RIS/PACS after obtaining Institution Review Board approval. The datasheets provided consisted of the subject’s code, age, gender, date of the US, name of the radiologist who performed the US scan, laterality of the US (right/ left/bilateral), and CSA measurement (mm^2^). EMG output (normal/mild/moderate/severe) was extracted from electronic records. Patients with median nerve trauma or neoplasm, as well as those who had US without EMG, were excluded. For patients with bilateral ultrasound wrist assessment, each hand was considered as a separate image to be compared with EMG.

Statistical analysis

**Figure 1:**
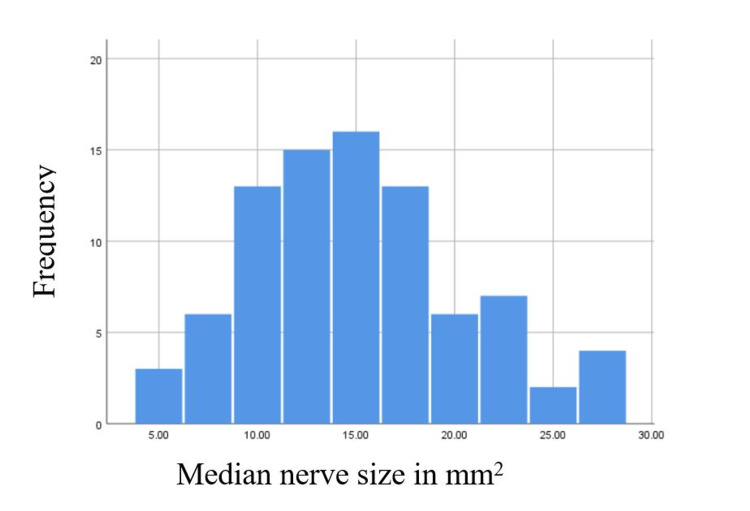
Bell-shaped histogram representing a normal distribution of US cross-sectional area (dependent variable), using SPSS 64-bit version 25.

Data were analyzed using SPSS 64-bit version 25 (IBM Corp., Armonk, NY). EMG findings were classified into normal, mild, moderate, and severe CTS subgroups (independent variable). The US CSA was used as the dependent variable. Histogram, Shapiro-Wilk, and Kolmogorov-Smirnov tests were used to assess distribution normality. The dependent variable had a value of 0.023 and 0.021 for the Shapiro-Wilk and Kolmogorov-Smirnov tests, respectively, indicating a normal distribution pattern. Figure 1 shows the histogram of the dependent variable. Levene's test was performed to assess the homogeneity of variance. Since our dependent variable is continuous and the independent variable has consisted of four categorical subgroups, the one-way analysis of variance (ANOVA) test and post hoc analysis were used to assess for difference after meeting other statistical assumptions. Because our independent variable subgroups were unequal in size, we specifically used the Scheffe Post Hoc test. US's sensitivity and specificity were calculated for CTS when compared to EMG (the gold standard). P-values of ≤0.05 were set as the cutoff for the results to be considered statistically significant.

Ethical considerations

Institutional Review Board approval was received from the Clinical Imaging Research Committee in HMC, reference number: CIRC-RDB-40-02-2021. Datasheets were coded to ensure patient anonymity. Access was given only to investigators with medical backgrounds during data cleansing and analysis. There was no investigator-patient interaction at any level. Datasheets were password protected. Analysis of the anonymous data was conducted on a computer-based system in a secured private area.

## Results

**Figure 2:**
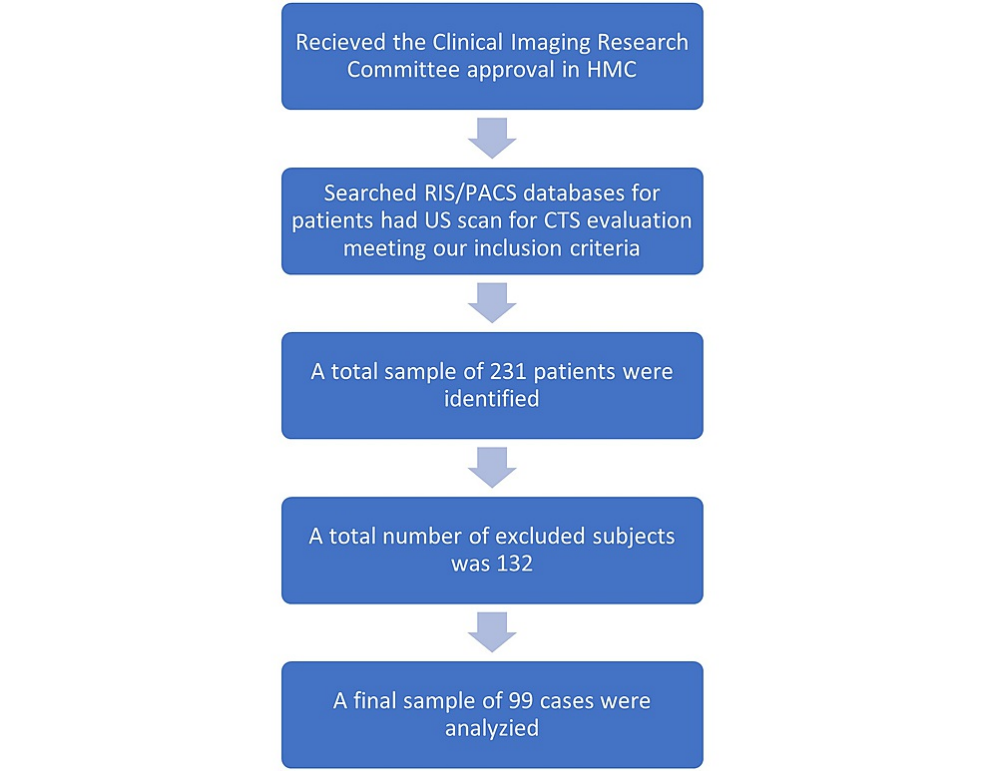
Flow chart demonstrates our study approach to reach the final sample.

**Figure 3:**
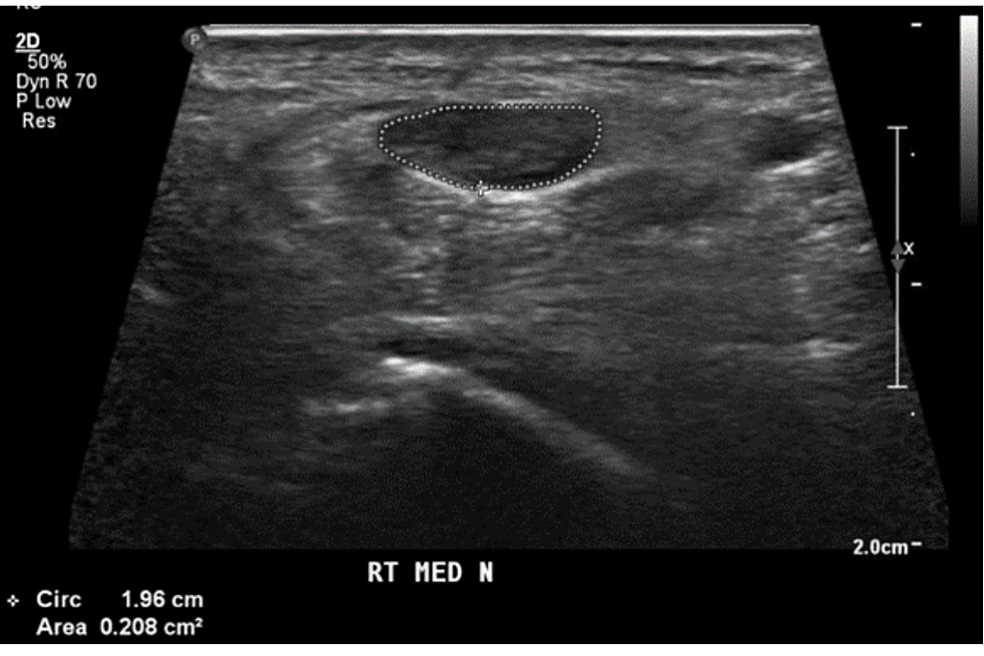
A transverse ultrasound view of the right wrist demonstrates the encircled surface area of the entrapped right median nerve with a cross-sectional area measuring 21 mm (2) representing severe carpal tunnel syndrome.

Our final sample consisted of 99 subjects: 81 females and 18 males. Figure 2 summarizes the steps conducted to reach the final sample. Their age range was between 24 and 87 years, with a mean of 49 ± 3 years. Fifty cases had bilateral US scans. Figure 3 illustrates how the CSA of the median nerve was measured for one of the US samples used in our study. Ten musculoskeletal radiologists performed US scans. The CTS classification using the median nerve CSA reported by the US when compared to EMG was as follows (95% confidence interval of the mean (CI)): normal: 8.7-10.8 mm^2^, mild CTS: 10.6-16.5 mm^2^, moderate CTS: 15.4-18.1 mm^2^, severe CTS: 19.1-23.1 mm^2^. There was a statistically significant difference between groups as determined by one-way ANOVA (p<0.001). A Scheffe Post Hoc test revealed no statistical difference between normal and mild CTS groups (p=0.08). There was a statistical difference between mild and moderate groups (p=0.006) and moderate and severe groups (p<0.001). Tables 1 and 2 demonstrate the outcomes of ANOVA and Scheffe Post Hoc tests. Our data showed US overall sensitivity for CTS of 73.8% and specificity of 76.3%.

**Table 1:** The outcomes of one-way ANOVA tests using SPSS 64-bit version 25.

	Sum of squares	df	Mean square	F	Sig.
Between groups	1510.701	3	503.567	47.482	0.000
Within groups	827.229	78	10.605		
Total	2337.930	81			

**Table 2:** The outcomes of Scheffe Post Hoc test using SPSS 64-bit version 25. *The mean difference is significant at the 0.05 level.

Scheffe Post Hoc multiple comparisons						
(I) EMG	(J) EMG	Mean difference (I-J)	Std. error	Sig.	95% confidence interval	
					Lower bound	Upper bound
Normal	Mild	–2.89583	1.09519	0.081	–6.0253	0.2337
	Moderate	–6.85737^*^	0.92185	0.000	–9.4916	–4.2232
	Severe	–11.5069^*^	1.01543	0.000	–14.4085	–8.6054
Mild	Normal	2.89583	1.09519	0.081	–0.2337	6.0253
	Moderate	–3.96154^*^	1.07956	0.006	–7.0464	–0.8767
	Severe	–8.61111^*^	1.16049	0.000	–11.9272	–5.2950
Moderate	Normal	6.85737^*^	0.92185	0.000	4.2232	9.4916
	Mild	3.96154^*^	1.07956	0.006	0.8767	7.0464
	Severe	–4.64957^*^	0.99855	0.000	–7.5029	–1.7962
Severe	Normal	11.50694^*^	1.01543	0.000	8.6054	14.4085
	Mild	8.61111^*^	1.16049	0.000	5.2950	11.9272
	Moderate	4.64957^*^	0.99855	0.000	1.7962	7.5029

## Discussion

This is the first study to investigate the role of US imaging in the evaluation of CTS in the state of Qatar. The role of the US in CTS evaluation is still under investigation due to variations among recommendations made by studies and to assure the external validity of some previously established studies.

Numerous studies were conducted worldwide to identify the ability of the US to diagnose and classify mild, moderate, and severe cases of CTS based on the CSA of the US. In this study, we found that CTS classification using the median nerve CSA reported by the US when compared to EMG was as follows (95% CI): normal: 8.7-10.8 mm^2^, mild CTS: 10.6-16.5 mm^2^, moderate CTS: 15.4-18.1 mm^2^, severe CTS: 19.1-23.1 mm^2^. Therefore, we suggest a median nerve CSA of ≤10 mm^2^ is more likely to correspond to a normal EMG. This cutoff was previously suggested by a study conducted in the United Kingdom [5]. Further, these ranges were consistent with a study conducted in Iran, where they had the following results: 12 ± 3 mm^2^ for mild, 15 ± 3 mm^2^ for moderate, and 19 ± 6 mm^2^ for severe CTS [4]. However, the ranges of the CSAs are statistically insufficient to withdraw conclusive results.

According to Kanikannan et al., the US was found to be highly sensitive to moderate and severe CTS but less sensitive to mild CTS [6]. This is consistent with our Scheffe Post Hoc finding of no statistical difference between normal and mild CTS groups (p=0.08), but the presence of a statistical difference between mild and moderate groups (p=0.006) and moderate and severe groups (p<0.001). Thus, Kanikannan et al. recommended choosing EMG over the US for clinically suspected mild cases [6].

Another important aspect of assessing the US profile is calculating the sensitivity and specificity for the diagnosis of CTS. A meta-analysis of 323 articles, over 3131 wrists, showed that the sensitivity and specificity of US for the diagnosis of CTS were 77.6% (95% CI 71.6-83.6%) and 86.8% (95% CI 78.9-94.8%), respectively [7]. Thus, our data’s sensitivity of 73.8% and specificity of 76.3% are consistent with the literature. Based on these findings, Flower et al. suggested that US may be a feasible alternative to EMG as the first-line confirmatory test; however, US may not replace EMG (gold standard) [7].

A few strengths and weaknesses in our study need to be highlighted. First, the strengths include that our CSA of the median nerve was captured by experienced radiologists in a single institution. Another advantage is the use of a different statistical approach than ones used in the literature (e.g., use of ANOVA, post hoc analysis) to determine the ability of the US to classify the severity of CTS cases based on CSA [6-10]. On the other hand, the main weakness is the small sample size, as most CTS cases are either diagnosed and managed clinically or have EMG only.

## Conclusions

In conclusion, sonography seems unable to differentiate some normal from mild CTS cases. However, a median nerve CSA of ≤10 mm2 is suggestive of normal EMG. Nevertheless, sonography appears to be excellent in diagnosing and classifying moderate and severe CTS cases. Identifying the severity of entrapment based on the US CSA of the median nerve may help radiologists classify cases during reporting; thus, it could influence the management and follow-up plan. More studies are needed to assure external validity.
